# Abnormal pelvic morphology and high cervical length are responsible for high-risk pregnancies in women displaying achondroplasia

**DOI:** 10.1186/s13023-016-0529-5

**Published:** 2016-12-05

**Authors:** Alexandre J. Vivanti, Anne-Gael Cordier, Geneviève Baujat, Alexandra Benachi

**Affiliations:** 1 Department of Obstetrics and Gynecology and Reproductive Medicine, AP-HP, Antoine Béclère Hospital, Univ Paris-Sud Clamart, Clamart, 92140 France; 2 Département de Génétique et INSERM U781, Université Paris Descartes-Sorbonne Paris Cité, Fondation Imagine, Hôpital Necker-Enfants malades, AP-HP, Paris, France

**Keywords:** Achondroplasia, High-risk pregnancy, Cervix, Ultrasound, Cesarean section

## Abstract

Pregnancies of women displaying achondroplasia are at high risk of adverse events. Early sonographic assessment of affected women can indicate an unusually long cervical length. It is the consequence of pathological anatomy of the pelvis. Thus, there is a foreseeable dystocia owing to cephalopelvic disproportion. Furthermore, this situation could also complicate cervical ripening prior to fetal extraction.

Dear Editors,

We would like to bring *Orphanet Journal of Rare Diseases* readers’attention to a specific high-risk pregnancy management. We report the case of a 30-year-old, gravida 1, para 0, with achondroplasia who became spontaneously pregnant. Clinical examination highlighted a pathological anatomy of the pelvis. Pelvic computed tomography showed marked shortening of the obstetric conjugate diameter (5.4 cm) (Fig. 1). In view of the patient’s disease, early sonographic assessment of the fetus was performed. An unusually long cervical length was observed: 61.7 mm at 16 weeks of gestation (Fig. 2). This measurement is way above the 95th centile (49.3 mm) [1].

**Fig. 1:**
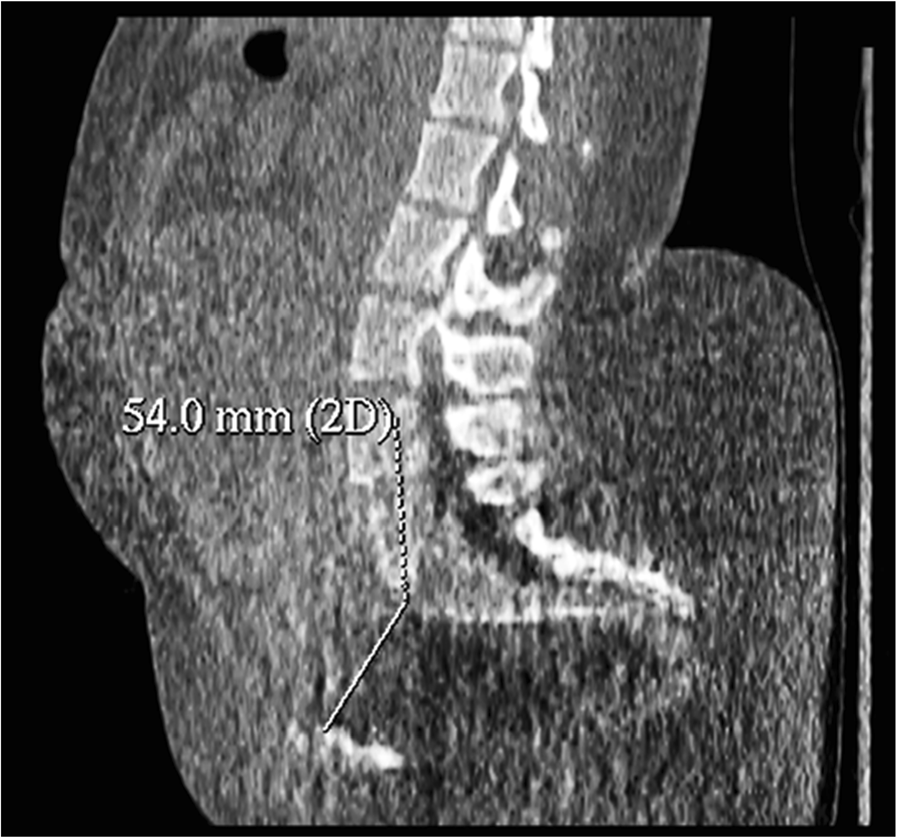
Pelvic computed tomography of a woman with achondroplasia. Sagittal image reconstruction of the pelvis of a woman with achondroplasia: the obstetric conjugate diameter is 5.4 cm (the normal value is 11 cm or more)

**Fig. 2:**
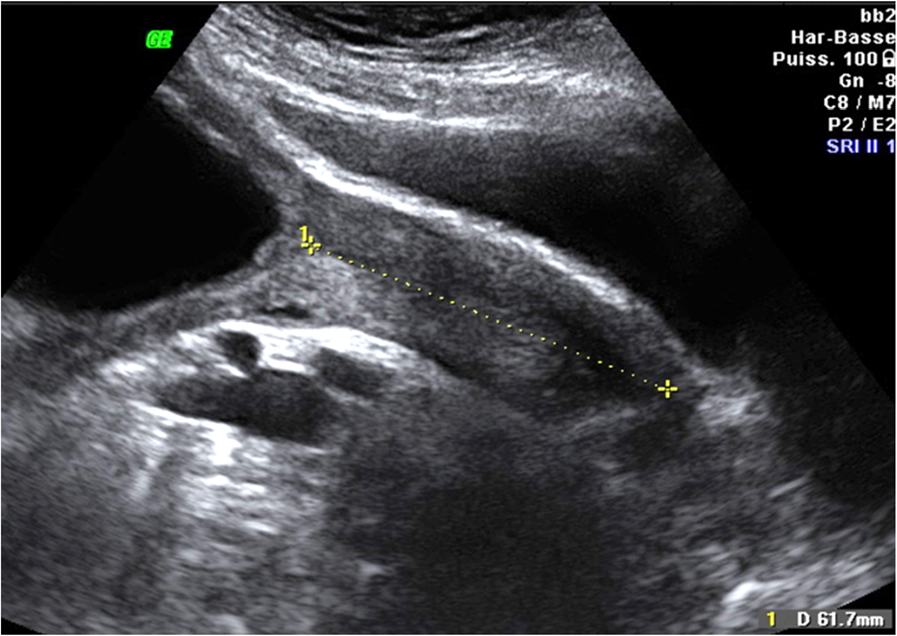
Sonographic measurement of cervical length in a woman with achondroplasia (16 weeks of gestation). Cervical length measurement using abdominal sonography of a woman with achondroplasia (16 weeks of gestation)

There are few data on the obstetric management of women with skeletal disorders such as achondroplasia or osteogenesis imperfect [2]. Achondroplasia is a genetic skeletal disorder with a prevalence of close to 1/20,000 pregnancies. It is characterized by a rhizomelic form of dwarfism with a prominent forehead and a low nasal bridge [3]. Although most affected women present premature ovarian failure, their fertility does not seem to be strongly affected, but not all obstetricians are used to managing such pregnancies. First, there is a need for routine cesarean section because of foreseeable dystocia owing to cephalopelvic disproportion. In the case of fetal demise or of termination of pregnancy requested because of an eligible malformation or an affected fetus, the abnormal pelvic morphology and biometry make vaginal delivery impossible when the biparietal diameter exceeds the obstetric conjugate, because of impaction resulting from lumbosacral hyperlordosis. Management of 1^st^ or early 2^nd^ trimester fetal demise is also risky. We noticed that women with achondroplasia have an abnormally high cervical length compared with unaffected women. This special feature, which may protect against preterm delivery, could also complicate cervical ripening prior to fetal extraction. The use of mechanical dilatation by osmotic cervical dilators could be compromised because of the abnormal cervical length, which could easily exceed the length of the mechanical device (impossibility to catheterize the internal orifice). If vacuum suction is planned, ultrasound guidance is needed in order to avoid cervical injury. We think that obstetricians should be aware of the risks associated with this specific pregnancy risk for women with achondroplasia. Termination of pregnancy in such cases warrants specific care to avoid high maternal morbidity.
